# Thermomechanical Studies of Yielding and Strain Localization Phenomena of Gum Metal under Tension

**DOI:** 10.3390/ma11040567

**Published:** 2018-04-07

**Authors:** Elżbieta A. Pieczyska, Michał Maj, Karol Golasiński, Maria Staszczak, Tadahiko Furuta, Shigeru Kuramoto

**Affiliations:** 1 Institute of Fundamental Technological Research, Polish Academy of Sciences, Pawińskiego 5 B, Warsaw 02-106, Poland; mimaj@ippt.pan.pl (M.M.); kgolasin@ippt.pan.pl (K.G.); mstasz@ippt.pan.pl (M.S.); 2 Toyota Central Research & Development Laboratories, Inc., Nagakute, Aichi 480-1192, Japan; e0646@mosk.tytlabs.co.jp; 3 Department of Mechanical Engineering, Ibaraki University; 4-12-1, Nakanarusawa, Hitachi 316-8511, Japan; shigeru.kuramoto.11@vc.ibaraki.ac.jp

**Keywords:** gum metal, yield limit, thermomechanical coupling, infrared thermography, digital image correlation, strain localization

## Abstract

This paper presents results of investigation of multifunctional β-Ti alloy Gum Metal subjected to tension at various strain rates. Digital image correlation was used to determine strain distributions and stress-strain curves, while infrared camera allowed for us to obtain the related temperature characteristics of the specimen during deformation. The mechanical curves completed by the temperature changes were applied to analyze the subsequent stages of the alloy loading. Elastic limit, recoverable strain, and development of the strain localization were studied. It was found that the maximal drop in temperature, which corresponds to the yield limit of solid materials, was referred to a significantly lower strain value in the case of Gum Metal in contrast to its large recoverable strain. The temperature increase proves a dissipative character of the process and is related to presence of *ω* and *α*″ phases induced during the alloy fabrication and their exothermic phase transformations activated under loading. During plastic deformation, both the strain and temperature distributions demonstrate that strain localization for higher strain rates starts nucleating just after the yield limit leading to specimen necking and rupture. Macroscopically, it is exhibited as softening of the stress-strain curve in contrast to the strain hardening observed at lower strain rates.

## 1. Introduction

Recently, a new class of beta-type Ti-based alloys called Gum Metal has drawn increasing attention due to its outstanding mechanical properties, i.e., a low Young’s modulus, high strength, a nonlinear superelastic-like large recoverable strain, high plastic performance, and workability without hardening [1]. Gum Metal is also characterized by Elinvar- and Invar-like behaviors, which guarantee its reliable application in a wide temperature range. The alloy is fabricated by powder metallurgy followed by solution treatment with cold working. Due to the unique performance, Gum Metal has been successfully applied in precision and sport industries. Furthermore, due to its high biocompatibility and low value of Young’s modulus, the alloy is a promising candidate for biomedical applications, e.g., implants and orthodontic wires [2,3]. Since the first publication on Gum Metal in English in 2003 [1], an increasing number of papers investigating the structural and mechanical features of the alloy under various loading conditions has been published [4,5,6,7,8,9]. The main research directions in several studies focus on the role of chemical composition, in particular oxygen content, which significantly influences the Gum Metal properties hindering stress-induced phase transformations, specifically during large recoverable strain [10,11,12,13,14]. The superelastic-like nature of Gum Metal, one digit higher in recoverable strain (≈2.5%) compared to other metallic materials (≈0.25%), has been stressed in the literature [12,13,14,15]. Most of the initially published papers report that Gum Metal’s unique elastic behavior is related to the micro elastic fields induced to the alloy structure during the cold-working process. Comprehensive structural studies published in subsequent papers confirmed likewise a presence of *ω* and *α*″ phases. It means that phase transformations also play an important role in the deformation mechanisms of Gum Metal. Recent papers cover more detailed investigations about the phenomena occurring during the plastic deformation of the alloy [16,17]. Gum Metal plasticity was initially believed to be governed by a “dislocation-free” giant fault mechanism [1,2,3,4]. However, the latest results demonstrate that the giant fault mechanism appears to be a phase-transformation-assisted nanotwinning mechanism. It governs Gum Metal plasticity without direct assistance from dislocations during the process [17]. Different aspects which influence Gum Metal’s properties can be studied, among others fracture toughness. For example, the characterization of the fracture toughness at small scales using a pillar-spitting technique can provide new results and be important in the design of new systems as was shown in [18].

Valuable information can be obtained from detailed analysis of the effects of thermomechanical couplings, i.e., the mechanical and thermal data, captured during the deformation process [11,12,13,19,20,21,22]. To the best of the authors’ knowledge, an analysis of nonlinear deformation, elastic-plastic transition, and localization phenomena in Gum Metal using coupled mechanical and thermal effects has not been reported so far. Therefore, the goal of the present paper is to discuss subsequent stages of Gum Metal’s deformation, i.e., the elastic stage and yielding phenomenon followed by plastic deformation, including nucleation and development of the strain localization, up to rupture. For this purpose, coupled mechanical and thermal fields were determined using infrared thermography (IRT) [23,24,25,26,27,28] and digital image correlation (DIC) [29,30]. The thermal response of Gum Metal under loading reveals a thermodynamic nature of the governing deformation mechanisms, which are still being discussed in the literature.

## 2. Materials and Methods

### 2.1. Material and Specimens

**Figure 1:**
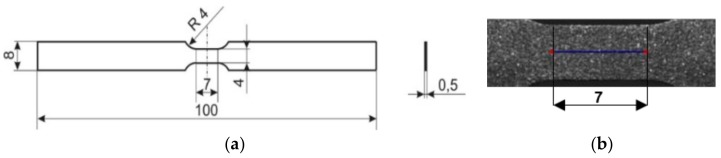
(**a**) Geometry and dimensions of Gum Metal specimen; (**b**) surface prepared for digital image correction (DIC) analysis with the denoted position of the virtual extensometer.

Gum Metal with composition of Ti–23Nb–0.7Ta–2.0Zr–1.2O (at. %) was provided by *Toyota Central Research & Development Laboratories Inc.* The fabrication procedure was comprised of powder metallurgy, sintering at 1300 °C for 16 h in a vacuum of 10^−4^ Pa, hot forging, solution treatment at 900 °C for 30 min, and subsequent quenching in water with ice. Then, the oxidized layer was removed and the material was cold worked in order to obtain high elastic and plastic properties. Finally, the fabricated Gum Metal was machined into flat specimens with the geometry and dimensions presented in Figure 1a. A specimen’s surface prepared for DIC analysis, showing the length and position of the virtual extensometer used in the experiment, is depicted in Figure 1b.

### 2.2. Determination of Mechanical and Thermal Fields Using DIC and IRT Techniques

**Figure 2:**
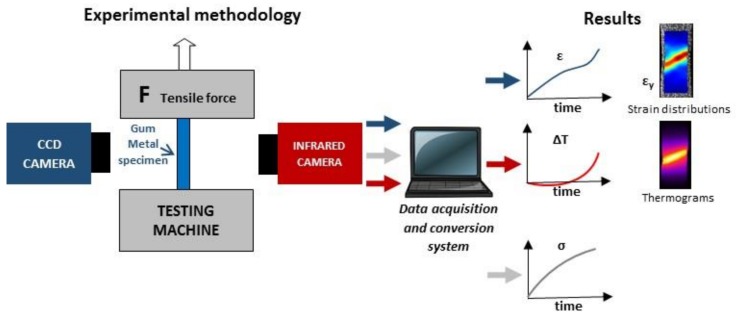
Experimental methodology for investigation of thermomechanical couplings in Gum Metal using DIC and infrared thermography (IRT) techniques. CCD = charge-coupled device.

A scheme and a picture of the experimental setup applied for investigation of thermomechanical couplings occurring in Gum Metal during tension are presented in Figure 2.

The setup consists of an MTS 858 testing machine and two cameras working in two different spectral ranges, i.e., in the visible range (0.3–1 μm) a Manta G-125B charge-coupled device (CCD) camera and in the infrared range (3–5 μm) a ThermaCam Phoenix IR camera.

A comparison of the main settings of both cameras used in the experiment is presented in Table 1. The cameras were placed on the opposite sides of the Gum Metal specimen. One side, observed using the visible range camera, was covered with a speckle pattern of paint with micrometer-size metal particles in order to perform DIC analysis (see Figure 1b). The other side, observed by the IR camera, was covered by soot to increase and make uniform the surface emissivity. The applied procedure guarantees highly accurate mechanical and temperature measurements.

The tests were conducted with three different displacement rates, 0.007 mm/s, 0.07 mm/s, and 0.7 mm/s, which for the given geometry of the specimen corresponded to the mean strain rates 1·10^−3^ s^−1^, 1·10^−2^ s^−1^, and 1·10^−1^ s^−1^, respectively. During the deformation process, the loading force as a function of time and two image sequences in the visible and infrared ranges were recorded. The displacement and strain distributions were obtained from the visible range image sequence using a digital image correlation algorithm implemented in ThermoCorr software developed in IPPT [30]. The mean strain values for the gauge length l_0_ = 7 mm of the specimens were determined on the basis of DIC results using a virtual extensometer in the loading direction (Figure 1).

The temperature field was obtained using the IR camera based on the distribution of infrared radiation from the specimen’s surface. The absolute temperature determination is possible only for the surface of known emissivity after the calibration procedure. In the present work, an emissivity coefficient of the soot equal to 0.95 was assumed. From the obtained temperature distribution, the mean temperature was determined with high thermal sensitivity, up to 0.02 °C. The temperature change *ΔT_mean_* denotes the difference between the mean value of the temperature calculated for the gauge part of the tested specimen at each instant of straining *T_mean_ (t)* and the mean temperature of the same area before the deformation *T_mean_ (t_0_)*:

(1) $$\Delta T_{mean}=T_{mean}(t)-T_{mean}(t_{0}).$$

The value of the mean temperature was calculated for the gauge part of the specimen defined in the reference configuration. After the coupling procedure of the DIC and IRT results is performed, the mean temperature of all material points in the defined region can be calculated at each instant of straining. Details of the space and time coupling procedure are described in [30].

The temperature data can be presented as a function of time, strain, stress, or other parameters. The obtained temperature changes during deformation of Gum Metal specimens under straining served to analyze effects of thermomechanical couplings. A description of the temperature determination procedure applied for investigation of a shape memory polymer was presented in [23].

## 3. Results

### 3.1. Macroscopic Mechanical Response of Gum Metal in Tension up to Rupture at Various Strain Rates

**Figure 3:**
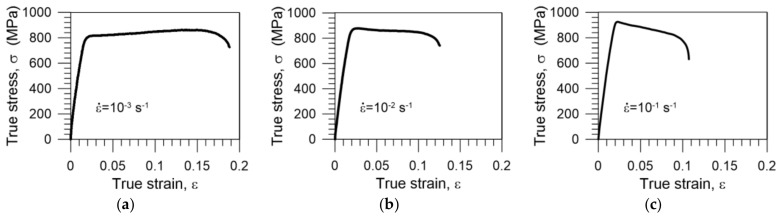
Stress-strain curves of Gum Metal subjected to tension until rupture at various strain rates: (**a**) 10^−3^ s^−1^; (**b**) 10^−2^ s^−1^; and (**c**) 10^−1^ s^−1^.

A comparison of the stress versus strain curves obtained for Gum Metal displacement-controlled tensile loading at strain rates of 10^−3^ s^−1^, 10^−2^ s^−1^, and 10^−1^ s^−1^ is presented in Figure 3. A clear effect of the strain rate on the regimes is noticed. At the higher strain rate, higher values of the maximal stress and lower values of the ultimate strain are observed. Furthermore, the character of the obtained stress-strain curve also depends on the strain rate: at the lowest strain rate 10^−3^ s^−1^, macroscopically observed stress-strain hardening is seen, at the strain rate 10^−2^ s^−1^, the curve is almost parallel to the strain axis, and at the highest strain rate, i.e., 10^−1^ s^−1^, a significant softening effect is noticed (Figure 3). However, irrespective of the strain rate, the characteristic features of the regimes, i.e.,: (i) linear elasticity; (ii) nonlinear superelastic-like recoverable deformation; (iii) the plastic stage; and (iv) damage, were obtained.

The area underneath the stress-strain curves contains information about the energy of the Gum Metal’s deformation, called toughness. The values of toughness estimated for the diagrams shown in Figure 3 are presented in Table 2.

The values of toughness change from 152 MJ∙m^−3^ for strain rate 10^−3^ s^−1^ to 81 MJ∙m^−3^ for strain rate 10^−1^ s^−1^ (Table 2). It means that the ability of the Gum Metal to absorb mechanical energy decreases as the strain rate increases.

Detailed thermomechanical analyses of the Gum Metal’s subsequent deformation stages will be the subject of the next sections.

### 3.2. Gum Metal Thermomechanical Behavior during Elastic-Plastic Transition

**Figure 4:**
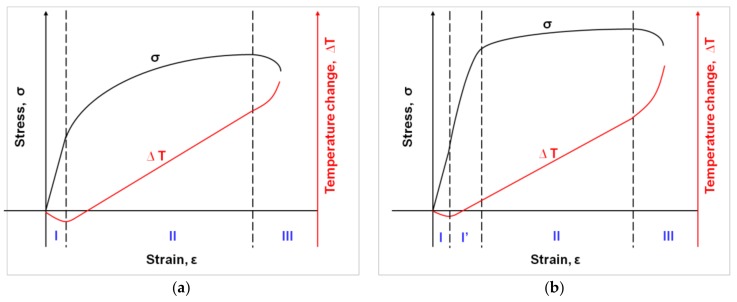
General scheme of stress *σ* and temperature change *ΔT* versus strain *ε* in tension up to rupture representing behavior of (**a**) any solid material, where subsequent deformation stages are distinguished: I = linear elastic, II = plastic, III = damage; (**b**) Gum Metal, where subsequent deformation stages can be distinguished: I = linear elastic, I’ = nonlinear mechanically reversible, II = plastic, III = damage.

Thermomechanical couplings play an important role during material loading and deformation. A solid material under loading can demonstrate endothermic, exothermic, or neutral behavior depending on the mode of the deformation process, its stage, and the material’s microstructure. The temperature changes accompanying the material’s deformation enable investigators to determine its yield point [19,20,21], analyze the nucleation and development of the strain localization [22,23,24], and estimate the energy storage [25]. Thus, the thorough determination of the effects of thermomechanical couplings can contribute to the knowledge about the behavior of new materials [20,23,26,30,31,32,33,34]. This section concerns analysis of the effects of thermomechanical couplings monitored in the initial stage of Gum Metal loading. A thermomechanical investigation of the elastic-plastic transition and recoverable strain is presented. The results were worked out and discussed for strain rates 10^−2^ s^−1^ and 10^−1^ s^−1^, since these tests were conducted close to adiabatic conditions (corresponding to the test durations 17.45 s and 1.67 s, respectively). The scheme of general dependence of the stress and temperature versus strain for solid material in tension is shown in Figure 4a, and that observed for Gum Metal in Figure 4b. The typical solid material subjected to tension in the elastic range gives a decreasing thermal response (a thermoelastic effect) (stage I), followed by its growth when plastic deformation begins (stage II), and finally its intense growth during localization and damage (stage III) (Figure 4a). Thus, as was studied by W. Thomson [19], Farren and Taylor [31], Taylor and Quinney [32], and Bever et al. [33], assuming adiabatic conditions, the stress corresponding to the lowest temperature can be associated to the yield limit, because the increase in temperature reveals the dissipative character of the deformation process, which is related to the permanent change in the material’s structure. The stress and temperature results formerly obtained for stainless steel, titanium alloy, polymers, etc. are in line with the scheme shown in Figure 4a and allowed us to indicate the material yield limit with a high accuracy [20,21,22,23]. In the case of Gum Metal, even the initial experiments conducted by the authors [24,34] showed that the maximal drop in temperature occurred significantly earlier (stage I) than the limit of its recoverable deformation (stages I and I**^’^**) as demonstrated in Figure 4b.

**Figure 5:**
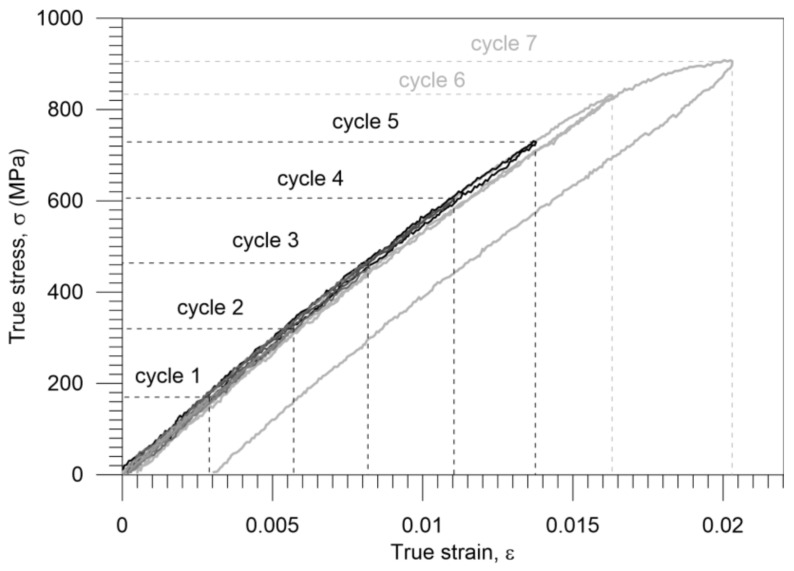
Stress versus strain for Gum Metal during incremental cyclic loading–unloading at a strain rate of 10^−2^ s^−1^ calculated from the DIC virtual extensometer; step 0.0025 for cycles 1–6 and 0.005 for cycle 7.

The strain value related to the alloy’s maximal drop in temperature was appointed by using the DIC algorithm in order to elucidate the observed behavior of Gum Metal. Moreover, the limit of the alloy’s mechanically reversible deformation was determined by an additional experiment composed of incremental loading–unloading tensile cycles with a small strain step. Stresses versus strain curves obtained for the seven subsequent cycles are presented in Figure 5. It was found that the mechanically reversible deformation was present up to cycle 5. Recoverable strain was equal to 0.0138 for the strain rate 10^−2^ s^−1^.

**Figure 6:**
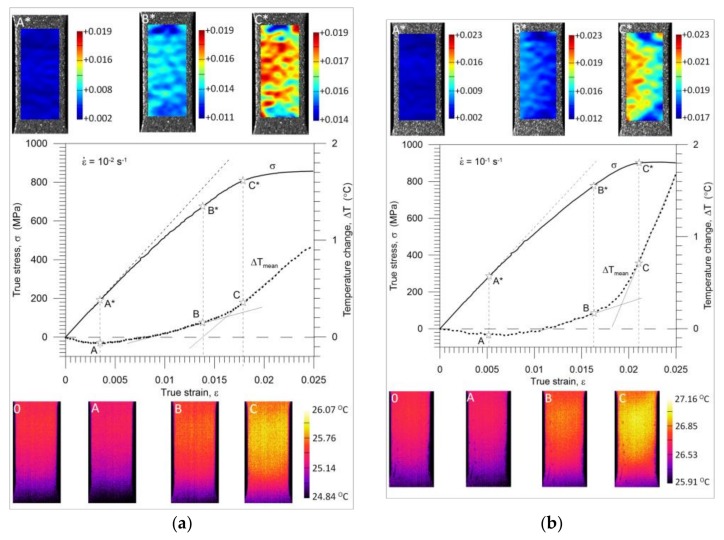
Stress *σ* and temperature change Δ*T_mean_* versus strain *ε* for Gum Metal’s tension in the initial deformation range at strain rates of (**a**) 10^−2^ s^−1^ and (**b**) 10^−1^ s^−1^ with strain distributions (above; A*-C*) and thermograms (below; 0, A-C).

The stress *σ* and mean temperature changes Δ*T_mean_* versus strain for the initial stage of tension at strain rates of 10^−2^ s^−1^ and 10^−1^ s^−1^ are shown in Figure 6a,b, respectively. In the same figures, strain distributions in the direction of tension ε_y_ obtained by the DIC algorithm and temperature distributions obtained by IRT are presented. The thermogram marked by 0 denotes the temperature distribution before specimen loading. The marked points A*-A, B*-B, and C*-C denote the stress and temperature values corresponding to the Gum Metal’s elastic limit (maximal drop in temperature), the limit of the mechanically reversible deformation, and the start of almost linear, significantly higher increase in temperature, respectively (Figure 6).

The thermal response in the initial loading range reaches its minimal value (points A-A*) significantly before the end of the nonlinear reversible deformation of Gum Metal (points B-B*) as shown in Figure 6a. Recoverable strain was determined in the incremental cyclic tension at 10^−2^ s^−1^ and is presented in Figure 5. Similar results were found at the higher strain rate of 10^−1^ s^−1^ as depicted in Figure 6b. The obtained stress-strain and the related temperature changes are in line with the scheme demonstrating Gum Metal’s thermomechanical behaviour in Figure 4b.

A comparison of the estimated values of characteristic deformation stages 0-A-B-C depicted in Figure 6a,b is presented in Table 3.

For the strain rate 10^−2^ s^−1^, the strain value related to the maximal drop in temperature equals 0.0035 (the related stress is 192 MPa), whereas the limit of reversible strain is equal to 0.0138. For the higher strain rate 10^−1^ s^−1^, the strain value related to the maximal drop in temperature is 0.0052 (the related stress is 285 MPa), whereas the limit of reversible strain is equal to 0.0163.

### 3.3. Nucleation and Development of the Strain Localization

**Figure 7:**
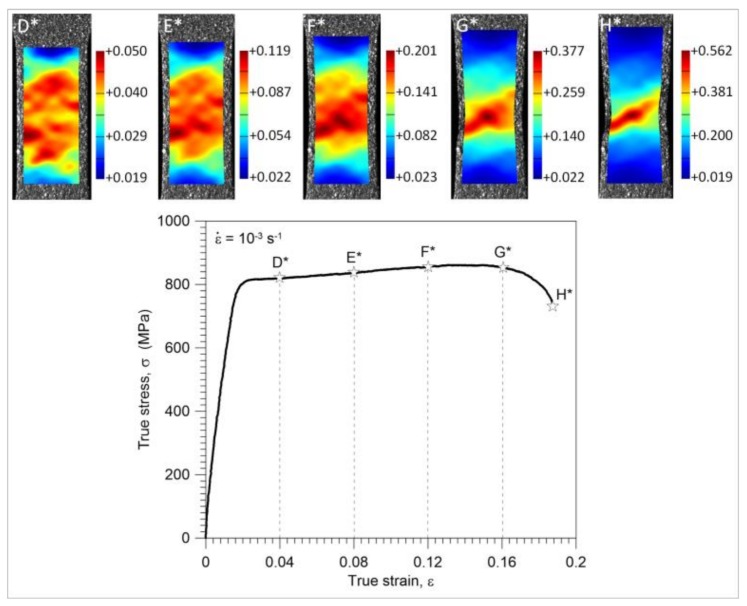
Stress versus strain completed by DIC distributions for Gum Metal during tension at a strain rate of 10^−3^ s^−1^.

Investigation of nucleation and development of the strain localization, leading to the Gum Metal specimen’s necking and damage, was also conducted on the basis of the results of the coupled thermomechanical fields obtained using DIC and IRT. Three different strain rates were considered; however, the thermal data were taken into account only for the higher strain rates 10^−2^ s^−1^ and 10^−1^ s^−1^, because after some initial tests it was found that the results obtained for the strain rate 10^−3^ s^−1^ were too much influenced by the heat exchange between the specimen and the surroundings. The stress versus strain curve for the tension at the strain rate of 10^−3^ s^−1^ up to the specimen’s rupture is shown in Figure 7. Above the diagram, the distributions of the strain component ε_y_ in the loading direction are presented at the values of the average strain equal to 0.04 (D*), 0.08 (E*), 0.12 (F*), 0.16 (G*), and just before the specimen’s rupture (H*).

**Figure 8:**
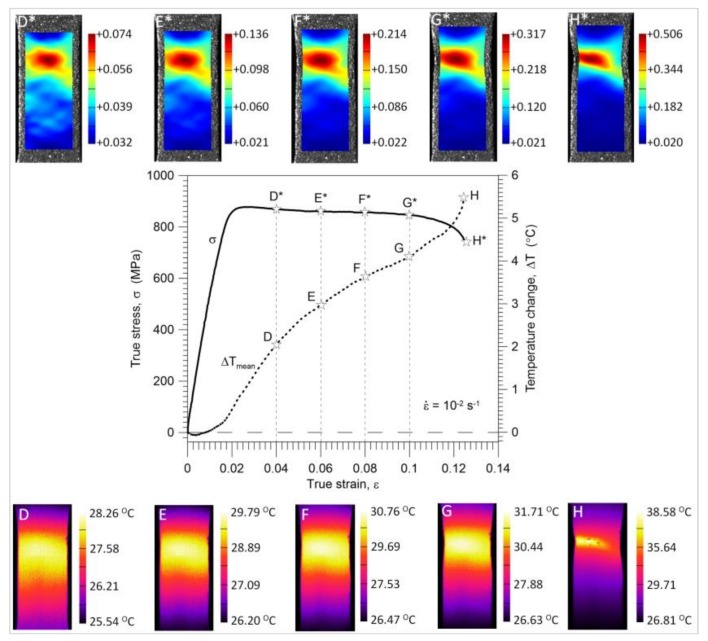
Stress *σ* and temperature change Δ*T_mean_* versus strain *ε* completed by related DIC strain (above) and IR temperature (below) distributions for Gum Metal in tension at a strain rate of 10^−2^ s^−1^.

**Figure 9:**
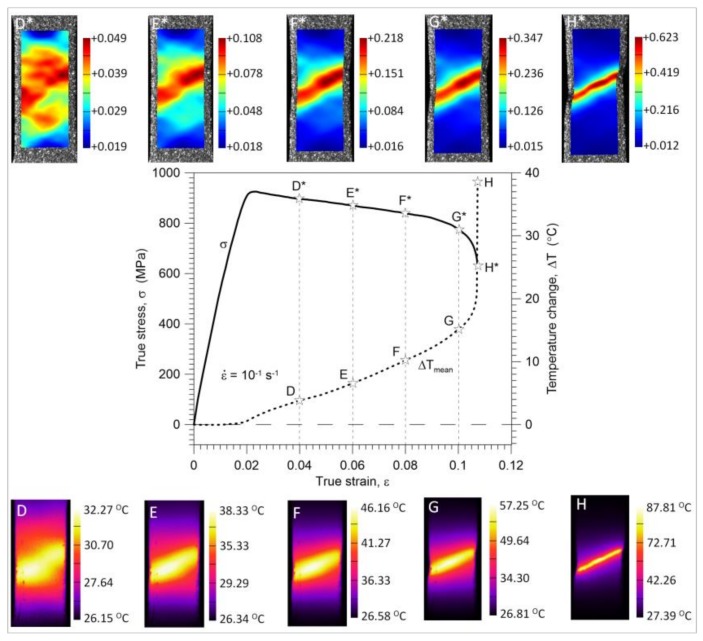
Stress *σ* and temperature change Δ*T_mean_* versus strain *ε* completed by related DIC strain (above) and IR temperature (below) distributions for Gum Metal in tension at a strain rate of 10^−1^ s^−1^.

It is seen that the strain distributions are macroscopically relatively uniform up to point E* (0.08 of a mean strain). After that, (point F*), the so-called ‘diffusive neck’ formation, is observed and further development of the strain localization up to the specimen’s rupture takes place. The stress and temperature change versus strain curves obtained for the strain rates of 10^−2^ s^−1^ and 10^−1^ s^−1^ are depicted in Figure 8 and Figure 9, respectively. In the same figures, the strain and temperature distributions are shown at the values of the average strain equal to 0.04 (D*-D), 0.06 (E*-E), 0.08 (F*-F), 0.10 (G*-G), and just before the specimen’s rupture (H*-H); 0.13 for the strain rate of 10^−2^ s^−1^ and 0.108 for the strain rate of 10^−1^ s^−1^.

The maximal temperature change related to the specimen’s rupture at the strain rate of 10^−2^ s^−1^ equals 5 °C, whereas that obtained at the strain rate of 10^−1^ s^−1^ equals 38 °C.

**Figure 10:**
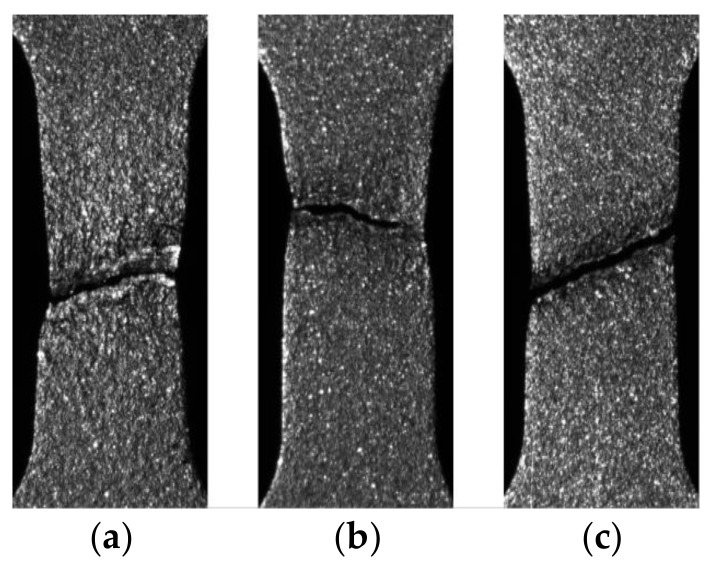
Images of specimens after rupture for strain rates: (**a**) 10^−3^ s^−1^; (**b**) 10^−2^ s^−1^; and (**c**) 10^−1^ s^−1^.

Images of specimens after rupture in the visible range for all tested strain rates are presented in Figure 10. It is seen that for the strain rates 10^−3^ s^−1^ and 10^−2^ s^−1^, the rupture zones demonstrate the same character. In both cases, significant necking is observed whereas for 10^−1^ s^−1^ that effect is much smaller. Moreover, for the strain rate of 10^−1^ s^−1^ the fracture profile is almost a straight line, whereas for the lower strain rates the profiles are more complex.

## 4. Discussion

A significant variation in the mean temperature change Δ*T_mean_* versus strain *ε* characteristics observed in the range of the mechanically reversible deformation (Figure 6a,b) includes an important message about the Gum Metal deformation process from a thermodynamic point of view. At the strain rates of 10^−2^ s^−1^ and 10^−1^ s^−1^, the maximal drops in temperature (points A) occur at true strain values of approximately 0.0035 and 0.0052, whereas the mechanically reversible strain limits (points B) at the strain values of approximately 0.0138 and 0.0163, respectively. The following increase in the specimen’s temperature, which starts from point A, reveals the initiation of the dissipative character of the deformation process. The dissipation observed between points A and B is caused by the exothermal-stress-induced phase transition of *ω* phase or *α*″ phase reported in the literature [14,15,16,17]. Furthermore, such a small increase in the temperature obtained in this range can be a sign that the phase transition takes place in a very small volume of the alloy, which is consistent with microstructural analyses [12,13,14,15]. At larger strains, a significant increase in temperature starts from point C. It means that plastic deformation becomes a dominant process at this stage, whereas the stage B-C is the transient period where both the phase transitions and plastic deformation in some areas of the specimen take place simultaneously. From point B, the deformation process is irreversible from both the thermodynamic and mechanical points of view. According to the field data, the temperature distributions obtained for loading points 0, A, B, and C are quite uniform for both considered strain rates whereas the strain fields demonstrate some non-uniformities, especially at the stage related to point C*, where plastic deformation is evident. The strain and temperature distributions shown in Figure 8 and Figure 9, respectively, represent more advanced stages of the deformation process in comparison to those depicted in Figure 6a,b. As shown in Figure 6, Figure 7, Figure 8 and Figure 9, the strain localization starts at point C* and proceeds from point D* up to the specimen’s rupture. In the case of the strain rate 10^−2^ s^−1^, the deformation is localized in the bigger area in comparison to the strain rate 10^−1^ s^−1^ (F*, G*, H*). As could be expected, the temperature distributions obtained for the strain rate of 10^−1^ s^−1^ presented in Figure 9 are much more distinct, since the test was conducted closer to adiabatic conditions. The changes of stress and related temperature are larger and the mean strain range to rupture is shorter at the higher strain rates (Figure 3, Figure 8 and Figure 9). Nevertheless, the maximal strain value calculated by the DIC algorithm for the strain rate of 10^−1^ s^−1^ (0.623) is higher than that observed for the strain rate of 10^−2^ s^−1^ (0.506). On the other hand, for the strain rate of 10^−3^ s^−1^, the calculated maximal strain value equals 0.562.

The analysis of the strain and temperature fields showed that the increase in the strain rate affects both the onset and development of the strain localization. The increase in the strain rate accelerates the strain localization process and causes the area where the strain is localized to be much smaller.

## 5. Conclusions

Subsequent stages of tensile loading of multifunctional Ti alloy—Gum Metal—were studied by analysis of coupled mechanical and thermal field data obtained by DIC and IRT techniques. The mechanical characteristics confirmed the Gum Metal’s large nonlinear superelastic-like behavior, low elastic modulus, and high strength, while the related temperature changes provided new thermodynamic data for analysis of the alloy’s elastic-plastic transition and development of the strain localization leading to the necking and rupture.

It was found that, irrespective of the applied strain rate, the maximal drop in the Gum Metal’s temperature (thermoelastic effect) occurs significantly earlier than the limit of its mechanically reversible deformation. The increase in the temperature between the maximal drop and mechanically reversible stage reveals a dissipative character of the deformation in this range. Due to the dissipative nature of the process, the large recoverable deformation of Gum Metal should be called “superelastic-like behavior” rather than “nonlinear elasticity”, which have been used interchangeably in the literature.

A large limit of the Gum Metal’s mechanically reversible nonlinear deformation, underlined as the new titanium alloy’s “super property”, is caused by stress-induced phase transformation of *ω* and *α*″ phases of exothermic character induced in the alloy’s structure during the technology process and activated during the loading process.

A significant effect of the strain rate on the macroscopic mechanical response of Gum Metal was observed: at the higher strain rates, higher values of maximal stress and lower values of ultimate strains were found. Furthermore, at the strain rate of 10^−3^ s^−1^, macroscopically observed stress-strain hardening was noticed. At the strain rate of 10^−2^ s^−1^, the curve was almost parallel to the strain axis. Yet, at the higher strain rate of 10^−1^ s^−1^, the strain localization process starts just after the onset of irreversible deformation, which was revealed macroscopically by a softening effect on the stress-strain curve.

The strain field analysis also demonstrated that the increase in the strain rate affects both the onset and development of the strain localization process. In the case of the strain rate of 10^−3^ s^−1^, the deformation is macroscopically uniform up to the mean strain value equal to 0.08, whereas for the strain rates of 10^−2^ s^−1^ and 10^−1^ s^−1^ the strain localization occurs at the earlier stage of the process and is localized in a smaller area, which was also confirmed by the temperature distribution.

## Figures and Tables

**Table 1:** Settings of visible and infrared cameras used in the experiment.

Cameras Properties	Manta G-125B	ThermaCam Phoenix
Resolution (pixel)	1100 × 410	320 × 150
Recording frequency (Hz) used for:		
10^−3^ s^−1^	2.93	5.86
10^−2^ s^−1^	14.57	116.6
10^−1^ s^−1^	58	583
Exposure/integration time (ms)	0.2	0.5
Pixel size (μm)	9.5	30

**Table 2:** Toughness values of Gum Metal determined during tension at three strain rates.

Strain Rate	10^−3^ s^−1^	10^−2^ s^−1^	10^−1^ s^−1^
Toughness values (MJ ∙ m^−3^)	152	97	81

**Table 3:** Comparison of estimated values of characteristic deformation stages (Figure 6a,b).

Strain Rate	Maximal Drop in Temperature (°C)	Strain at Minimal Temperature	Stress at Minimal Temperature (MPa)	Temperature at Reversible Deformation (°C)	Strain at Reversible Deformation	Stress at Reversible Deformation (MPa)
10^−2^ s^−1^	−0.062	0.0035	192	+0.15	0.0138	674
10^−1^ s^−1^	−0.071	0.0052	285	+0.22	0.0163	780
